# Tissue expression and antibacterial activity of host defense peptides in chicken

**DOI:** 10.1186/s12917-016-0866-6

**Published:** 2016-10-13

**Authors:** Mi Ok Lee, Hyun-Jun Jang, Deivendran Rengaraj, Seo-Yeong Yang, Jae Yong Han, Susan J. Lamont, James E. Womack

**Affiliations:** 1 WCU Biomodulation Major, Department of Agricultural Biotechnology, Seoul National University, Seoul, Korea; 2 Department of Veterinary Pathobiology, Texas A & M University, College Station, TX USA; 3 College of Pharmacy, Dankook University, Cheonan, Korea; 4 Department of Animal Science, Iowa State University, Ames, IA USA

**Keywords:** Host defence peptides (HDPs), Antimicrobial, Chicken

## Abstract

**Background:**

Host defence peptides are a diverse group of small, cationic peptides and are important elements of the first line of defense against pathogens in animals. Expression and functional analysis of host defense peptides has been evaluated in chicken but there are no direct, comprehensive comparisons with all gene family and individual genes.

**Results:**

We examined the expression patterns of all known *cathelicidins*, *β-defensins* and *NK-lysin* in multiple selected tissues from chickens. *CATH1* through 3 were predominantly expressed in the bone marrow, whereas *CATHB1* was predominant in bursa of Fabricius. The tissue specific pattern of *β-defensins* generally fell into two groups. *β-defensin1-7* expression was predominantly in bone marrow, whereas *β-defensin*8-10 and *β-defensin*13 were highly expressed in liver. *NK-lysin* expression was highest in spleen. We synthesized peptide products of these gene families and analysed their antibacterial efficacy. Most of the host defense peptides showed antibacterial activity against *E.coli* with dose-dependent efficacy. *β-defensin*4 and *CATH3* displayed the strongest antibacterial activity among all tested chicken HDPs. Microscopic analyses revealed the killing of bacterium by disrupting membranes with peptide treatment.

**Conclusions:**

These results demonstrate dose-dependent antimicrobial effects of chicken HDPs mediated by membrane damage and demonstrate the differential tissue expression pattern of bioactive HDPs in chicken and the relative antimicrobial potency of the peptides they encode.

**Electronic supplementary material:**

The online version of this article (doi:10.1186/s12917-016-0866-6) contains supplementary material, which is available to authorized users.

## Background

Bacterial infections in chickens are important not only for the health and productivity of the animals but also as a reservoir of foodborne human pathogens such as *Salmonella enterica*. Innate immunity is important in controlling bacterial infection, particularly at mucosal surfaces such as the gastrointestinsal, respiratory and reproductive tracts. Innate immune agents include antimicrobial secretions such as lysozyme, mucocilliary clearance, the acid environment of the gizzard and proventriculus and tight cellular junction at epithelial layers [1].

Host defense peptides (HDPs) are a diverse group of small, cationic peptides present in A wide variety of organisms including both animals and plants [2–6]. HDPs are an important first line of defense, particularly in those species whose adaptive immune system is lacking or primitive. A majority of HDPs are strategically synthesized in the host phagocytic and mucosal epithelial cells that regularly encounter microorganisms from the environment. Mature HDPs are broadly active against Gram-negative and Gram-positive bacteria, mycobacteria, fungi, viruses and even cancerous cells [7–9]. Several classification schemes have been proposed for AMPs; however, most AMPs are generally categorized into four clusters based on their secondary structures: peptides with a linear α-helical structure [10–12], cyclic peptides with a β-sheet structure [13–17], peptides with a β-hairpin structure [18], and peptides with a linear structure [19, 20].

It has become clear that HDPs are important and significant components of host defence against infection. The killing of bacteria appears to be very fast, ranging from 10 to 30 min for killing of *S. enteritidis* [21] and 30–60 min for killing of *E.coli* [22]. We have demonstrated chicken NK-lysin to destroy *E. coli* cell membranes within 5 min [23]. Furthermore, HDPs kill bacteria primarily through physical electrostatic interactions and membrane disruption. Therefore, it is difficult for microbes to gain resistance to HDPs [7, 24]. At the same time, most HDP’s have the capacity to recruit and activate immune cells and facilitate the resolution of inflammation [24, 25]. Therefore, it is not easy to differentiate therapeutic potential of HDPs, particularly against antibiotic-resistance bacteria. A highly promising approach to overcome drug resistance is to explore and exploit the huge diversity of innovative bioactive-engineered molecules provided by nature to fight pathogens. These include HDPs, natural products involved in the defense systems. So, with their obvious potential as novel therapeutic agents, understanding the HDPs, including the relationships between structure and mode of action of these molecules, is essential for the development of novel peptide-based antibiotics and immunotherapeutic tools.

Three major groups of HDPs, namely *cathelicidins (CATH)*, *defensins* and *NK-lysin* are present in vertebrate animals. *Defensins* constitute a large family of small, cysteine rich, cationic peptides that are capable of killing a broad spectrum of pathogens [26–29]. Vertebrate *defensins* are classified into three subfamilies, the α-, β-, and *θ-defensins*, characterized by different spacing of the six conserved cysteines. *Cathelicidins* are recognized by the presence of cathelin-like domains. The signal peptide and cathelin-like domains are well conserved across species, but the mature peptide sequences at the C-terminal regions are highly diverse [30]. Whereas the *defensin* structure is based on a common β-sheet core stabilized by three disulfide bonds [2], *CATH* s are highly heterogeneous. *NK-lysin* is a member of the saposin-like protein (SALIP) family, and is orthologous with human *granulysin* with a α-helical structure [9].

The first avian HDPs discovered were *β-defensins* from chicken and turkey, reported in the mid 1990-’s [31], and increasing information about HDPs in other avians species is becoming available [32]. The sequencing of the chicken (*Gallus gallus*) genome revealed the presence of a cluster of 14 different genes on chromosome 3 coding for avian *defensins (AvBD)* and designated then as *AvBD*1 to -14 [33, 34] and 4 *CATHs* densely clustered at the proximal end of chromosome 2 [35, 36]. *NK-lysin* was recently mapped to the distal end of chromosome 22 [37].

The highly inbred Leghorn Ghs-6 line has been used in many studies of immune function, including serving as a parental line of an advanced intercross line used to identify the association of genetic variants in the *AvBD* gene cluster with colonization of the cecum with *Salmonella enterica* serovar Enteritidis [38]. The bursa of Fabricius, a specialized immune organ in birds, arises from bursal epithelial cells around embryonic day 4, reaches a maximum size at 6–12 weeks after hatching [39] and previously demonstrated high expression of several of AvBDs [34]. The gene expression and antibacterial efficacy of all four *CATH* and several *AvBDs* has been evaluated individually, but there are no reports comparing the full spectrum of tissue expression and antimicrobial activity of chicken HDPs concordantly. Here, we have examined expression patterns of 14 *AvBD*, 4 *CATH* and *NK-lysin* with the highly inbred Leghorn Ghs-6 line and compared the antimicrobial activity of the peptides encoded against *E.coli.* Morphological change of *E.coli* membranes by *CATH* peptide treatment was also examined.

## Methods

### Birds

Chicks of the highly inbred Leghorn Ghs-6 line were produced and maintained in the Poultry Genetics Program at Iowa State University (Ames, IA). Birds were raised in light- and temperature-controlled pens with wood-shaving bedding and continual access to water and food meeting all NRC nutritional requirements. At 7 weeks of age, birds were euthanized according to the approved Institutional Animal Care and Use Committee protocol (Log #4-03-5425-G) and tissues immediately dissected. Bursa of Fabricius, thymus, spleen, bone marrow, cecal tonsil, duodenal loop, and liver tissue were collected. Samples consisting of either the entire tissue, or sections totalling approximately 1.0 cubic cm from larger tissues were harvested. The cecal tonsil included the lymphoid aggregates and surrounding tissue at the intersection of the two ceca and the gastrointestinal tract. Bone marrow was collected by expressing the marrow from both tibias of each bird with a narrow sterile wooden rod. Tissues were placed into RNAlater until used for isolation of mRNA.

### RNA extraction and quantitative reverse-transcription polymerase chain reaction (RT-PCR)

RNA extraction was performed using RNeasy Mini Kit (Qiagen) according to the manufacturer’s instructions. Total RNA samples were extracted from 5 birds tissues and used as a template for reverse transcription. cDNA was obtained by reverse transcriptase *SuperScript® III First-Strand Synthesis System* using 2 μg total RNA. The relative abundance of mRNA from genes was assessed by real time reverse-transcription (RT)-PCR using a Lightcycler 480 (Bio-Rad) and a Lightcycler 480 SYBR Green I master (Bio-Rad). Primer pairs specific for the amplification of *AvBD*, *cathelicidin* and *NK-lysin* genes are shown in Additional file 1: Table S1. PCR products were subjected to melt curve analysis and sequenced to confirm amplification of the correct gene. Data were analyzed by ddCt method. The mean threshold cycle value (Ct) of each sample was normalized to the internal control, *GAPDH*, and the expression profiles were obtained by comparing normalized Ct value with the calibrator sample, in which the gene exhibited the lowest expression level. Each analysis was performed in triplicate. Quantification of each sample was calculated with the cycle threshold values and standard curve information using the Lightcycler 480 version 1.5.0 software.

### Peptide synthesis

Nineteen synthetic linear peptides (Table 1) corresponding to chicken *defensins*, *cathelicidins* and *NK-lysin*, were synthesized and purified to >95 % purity grade through reverse-phase high-pressure liquid chromatography (Abclon, Seoul, Korea). Lyophilized peptide (1 mg each) was stored in desiccant at −20 °C and dissolved in phosphate buffer (pH 7.2) before use.

### Cell viability analysis of *Escherichia coli* after treatment with peptides

Gram-negative bacteria, *Escherichia coli* ATCC 25922, were purchased from Korean Collection for Type Culture and tested against each peptide. Cell viability analysis was carried out as previously reported [23]. Briefly, 6 × 10^6^ colony–forming units (CFU)/ml bacteria suspensions (90 μl) were placed into 96 well plates, followed by the addition of 10 μl of serial diluted peptide (final 0, 0.5, 1, 2.5, 5 μM) in triplicate. After 2 h incubation at 37 °C, equal volume of BacTiter-Glo™ Reagent (Promega) was added, and incubated for 5 min after which luminescence was measured with GloMax-Multi Detection System (Promega).

### Detection of damaged E. coli membranes after treatment with synthetic cathelicidin peptides

To visualize *E. coli* membrane damage, 6.5 × 10^6^ CFU of *E. coli* were incubated with 5 μM CATH1, CATH2, CATH3, or CATHB1 in 10 mM phosphate buffer (pH 7.0), respectively, at 37 °C for 2 h, and the membranes were observed by confocal laser scanning microscopy (Carl Zeiss, Oberkochen, Germany) after staining with the LIVE/DEAD BacLight bacterial viability kit (Invitrogen) according to the manufacturer’s protocol.

### Scanning electron microscopic analysis

To visualize membrane damages of *E. coli*, 6.5 × 10^6^ CFU of *E. coli* were incubated with 5 μM CATH1, CATH2, CATH3, or CATHB1 peptides at 37 °C for 5 min. After incubation, the bacteria were fixed with 2 % glutaraldehyde, washed, mounted, and damaged membranes were observed by a Field-Emission Scanning Electron Microscope (Carl Zeiss Inc.).

### Statistical analysis

GraphPad prism software was used for cell viability data analyses and gene expression analysis and data were expressed as mean ± SD. Statistical significance between groups or conditions was analysed by two-way or one-way ANOVA followed by Bonferroni’s *post hoc* test unless stated otherwise. Differences were considered to be statistically significant when *p* < 0.05.

## Results

### Tissue expression patterns

**Fig. 1:**
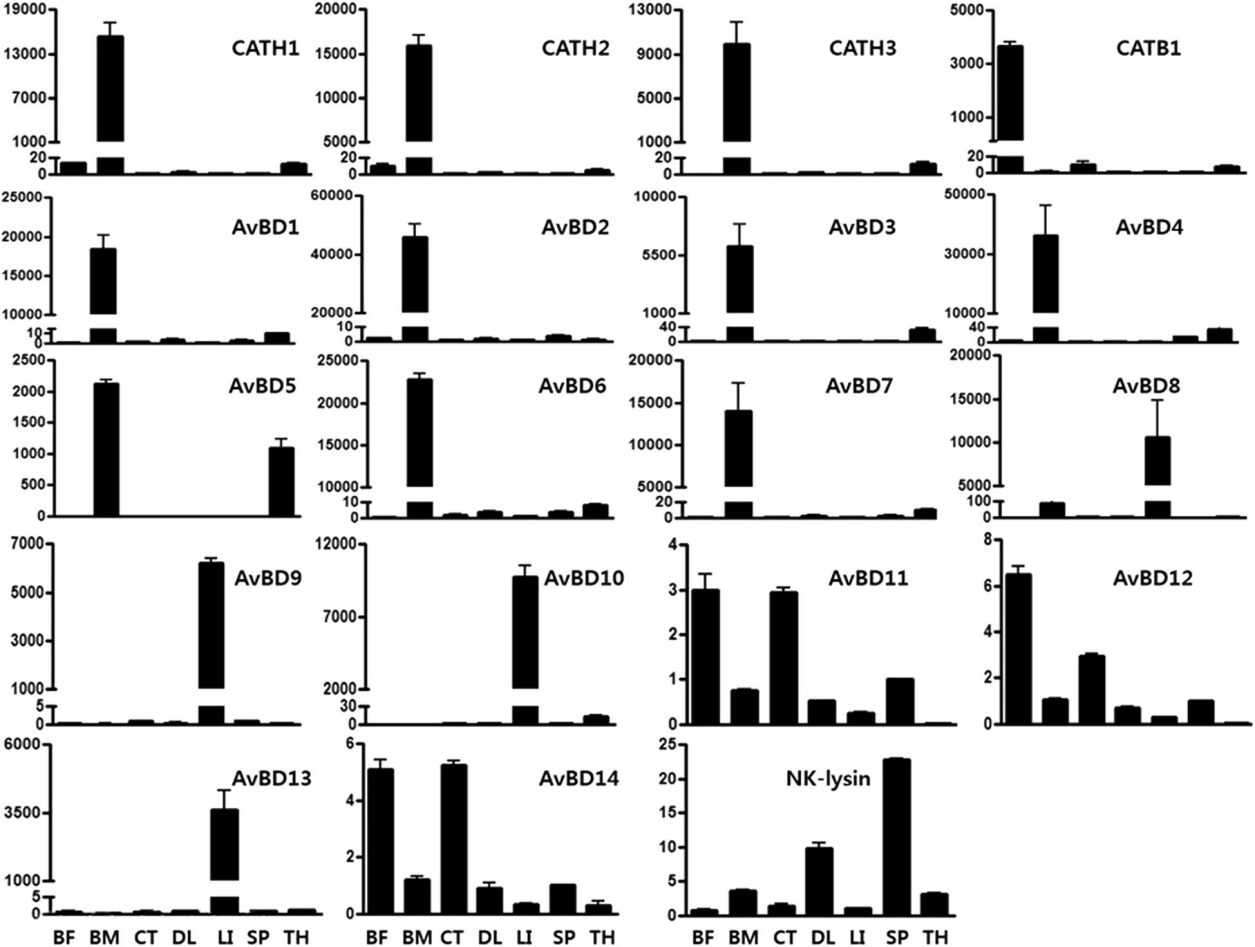
Tissue expression of chicken HDPs. Quantitative RT-PCR analysis of *CATH*, *AvBD* and NK-lysin gene expression in chicken tissues. The relative abundance of mRNA was assessed by normalization and calibrated to *glyceraldehyde 3-phosphate dehydrogenase* (*GAPDH*). *BF* Bursa of Fabricius, *BM* bone marrow, *CT* cecal tonsil, *DL* duodenal loop, *LI* liver, *SP* spleen and *TH* thymus

Quantitative RT-PCR was performed to examine the expression patterns of *CATH*, *AvBD* and *NK-lysin* genes in various chicken tissues. The chicken *AvBD*gene family has a unique expression pattern. *cAvBD1* through 7 are predominantly expressed in bone marrow and weakly expressed in thymus. *AvBD5* is an exception with strong expression in the thymus. The other *AvBDs*, *AvBD8* through 10 and *AvBD13* are predominantly expressed in liver. *AvBD11*, *AvBD12* and *AvBD14* are expressed in all tissues tested (Fig. 1). Chicken *CATH1*, *-2*, and *-3* are predominantly expressed in the bone marrow and to a lesser extent in bursa of Fabricius and thymus. *CATHB1*, however, showed abundant expression in bursa of Fabricius with low levels of expression in thymus and cecal tonsils. *NK-lysin* was predominantly expressed in spleen and in the duodenal loop, with lesser expression in thymus and bone marrow.

### Antimicrobial activity of chicken HDPs

**Fig. 2:**
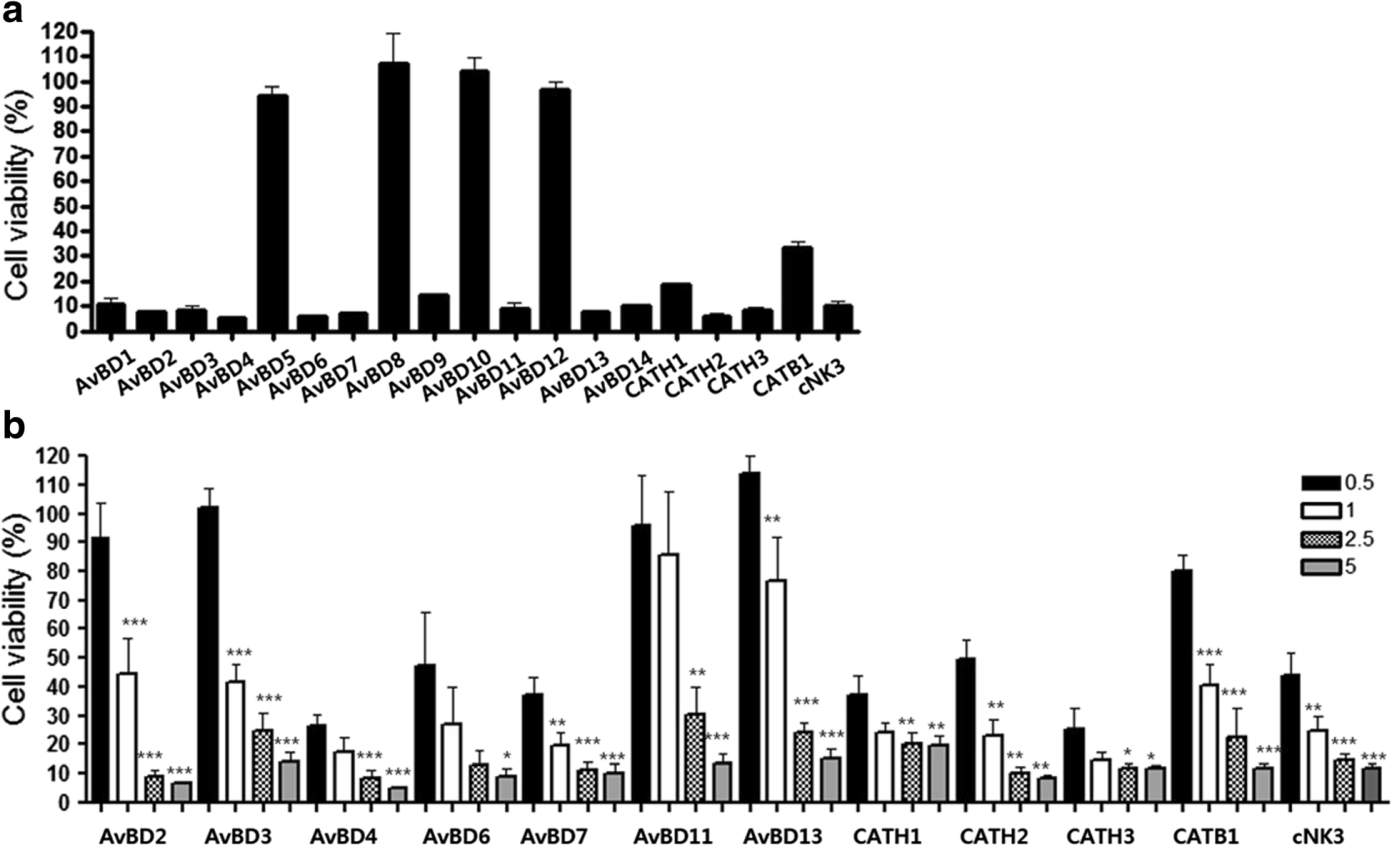
Antimicrobial activity of chicken HDPs against *E.coli. E. coli* (6 × 10^6^) were incubated with indicated amounts of peptide for 2 h and cell viability was compared to untreated cells. Nineteen chicken HDPs were tested under 5 μM concentrations (**a**) and 12 HDPs that showed more than 90 % killing activity in 5 μM treatment were selected for assay at lower concentrations (0.5, 1, 2.5, 5 μM) (**b**). Each *bar* represents the mean ± S.D. value of at least three independent experiments. Non-treated cell considered as 100 % cell viability and peptides treated cell viability was compared and calculated in percentile. *** indicate *p* ≤ 0.001, ** indicate *p* ≤ 0.01 and * indicate *p* ≤ 0.05

To address and compare the relative antibacterial activity of chicken HDPs, 14 *AvBD*, 4 *CATH* and one *NK-lysin* peptide were synthesized and tested for antimicrobial activity as previously described [23]. Most of the tested HDPs showed strong antibacterial activity against *E.coli* (Fig. 2a)*,* but 4 peptides AvBD5, -8, -10 and -12, showed very weak lytic activity at 5 μM. The majority of the peptides, however, killed more than 80 % of *E.coli* under test conditions.

We selected peptides that exhibited strong antimicrobial activity at 5 uM (less than 15 % survival rate), and tested antibacterial activity with lower concentrations of peptide (0.5, 1, 2.5 and 5 μM). These HDPs killed bacteria in a dose-dependent manner. Most peptides produced less than 20 % bacterial survival at low micromolar concentration (2.5 μM) and AvBD4, AvBD6, AvBD7, CATH1, CATH2, CATH3 and cNK3 show very strong antibacterial activity at all concentration (Fig. 2b)*.* These peptides killed 50 % of bacteria at very low (0.5 μM) micromolar concentration. AvBD4 and CATH3 displayed the strongest antibacterial effect among all tested chicken HDPs under test conditions. These results suggest that all functional peptides from chicken HDP had effective antibacterial activities under a broad range of peptide concentrations.

### Membrane damage by synthetic peptides

**Fig. 3:**
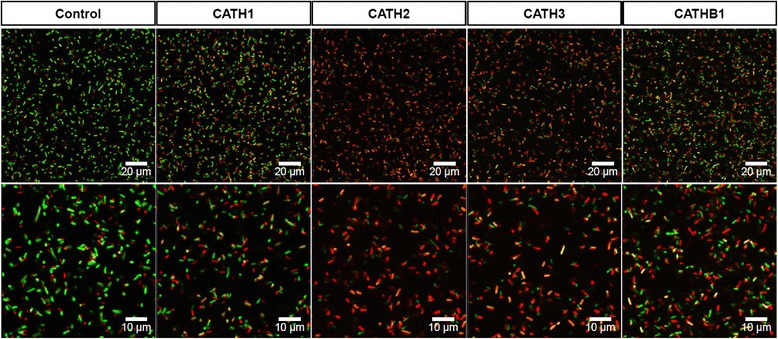
*Escherichia coli* viability and membrane damage following treatment with cathelicidins. Membrane damage in *E. coli* treated with each synthetic cathelicidin peptide (5 μM) was detected with a LIVE/DEAD BacLight Bacterial Viability Kit. *Green fluorescence* indicates live bacteria with intact membranes; *red fluorescence* indicates dead bacteria with damaged membranes. *CATH1* cathelicidin1, *CATH2* cathelicidin2, *CATH3* cathelicidin3, and *CATHB1* cathelicidinB1

To determine whether chicken HDPs altered the morphology and viability of *E. coli,* we used four *cathelicidin* peptides that have strong antimicrobial activity (Fig. 2). The damage to *E. coli* cell membranes after treatment with peptide was determined with confocal laser scanning microscopy. In the absence of peptide, most of the *E. coli* cells were stained green, indicating an intact membrane. In the presence of 5 μM peptide treatment, the majority of *E. coli* cells were stained red, indicating membrane damage (Fig. 3). The membrane damage was greater with CATH2 or CATH3 than with CATH1 or CATHB1, consistent with dose-dependent cell killing data.

**Fig. 4:**
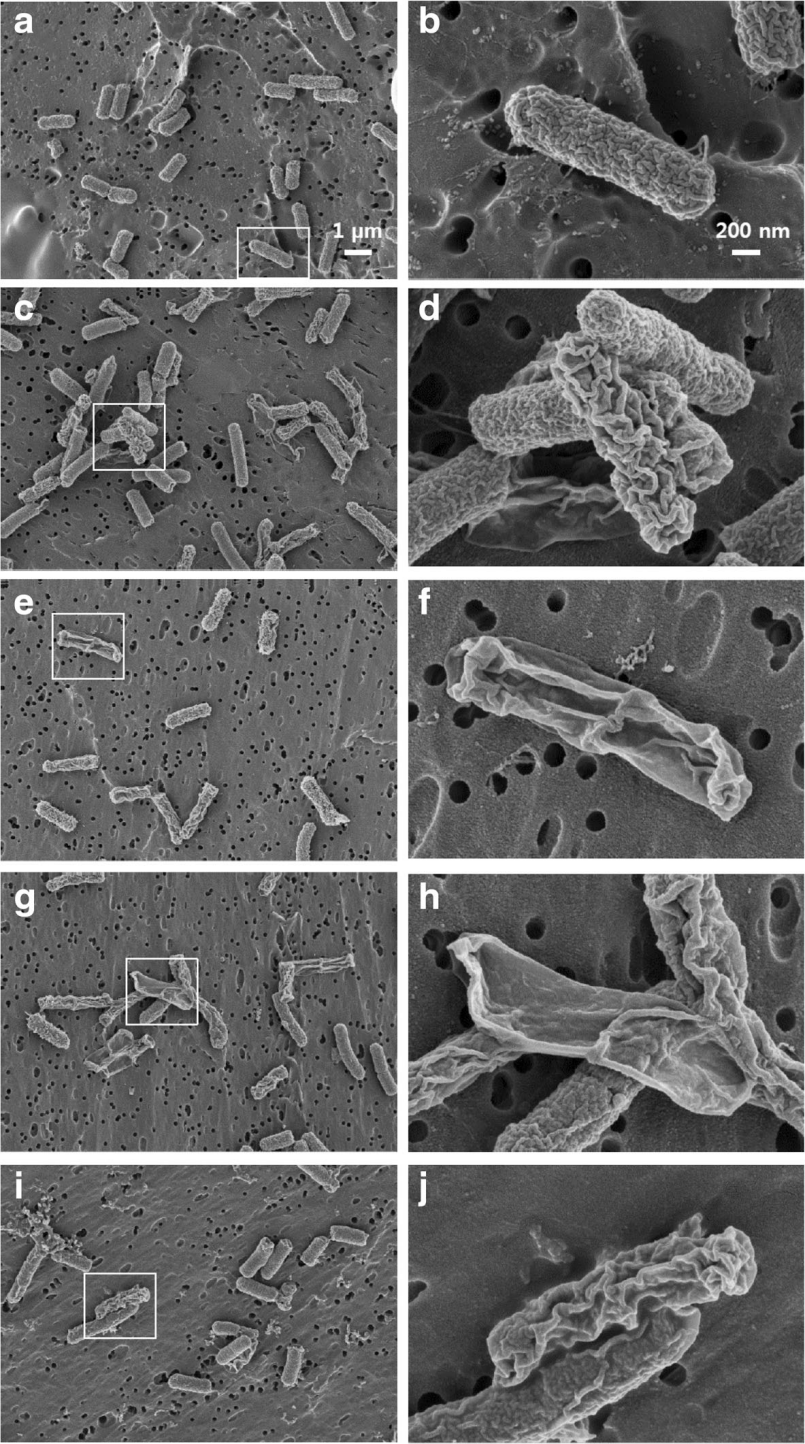
Scanning electron micrographs of cathelicidin-induced cell membrane damage. Scanning electron micrographs of *E. coli* with no treatment (control, **a** and **b**) or treated with cathelicidin1 (CATH1, **c** and **d**), cathelicidin2 (CATH2, **e** and **f**), cathelicidin3 (CATH3, **g** and **h**), or cathelicidinB1 (CATHB1, **i** and **j**). The *rectangle* as shown in the large-scale image

Scanning electron microscopy showed that untreated *E. coli* cells had normal, intact shape and uniform membrane surface (Fig. 4a and b). However, after treatment with each *CATH* an obvious difference in morphology was observed (Fig. 4c–j). The treated cells showed shrinkage, were rumpled, and lost their regularly arranged surface layer. The burst and crushed appearing cells were surrounded by debris. These findings suggest that *cathelicidin* destroy bacterial cells via membrane damage.

## Discussion

The aim of this study was to enhance understanding of tissue expression and antibacterial activity of chicken HDPs and to determine whether chickens can be a source of bioactive HDPs. As an initial step to understand HDPs, we examined their tissue expression profile, mainly in immune organs such as bursa of Fabricius, bone marrow, spleen and thymus. Cecal tonsil, duodenal loop and liver were also tested. Like their mammalian counterparts, avian cathelicidins and *β-defensins* are derived from bone marrow and/or epithelial cells. Chicken cathelicidins *CATH1*, *-2* and *-3*, are highly expressed in bone marrow with little to no expression in other tissues. In agreement with their myeloid origin*, CATH1-3* mRNA has been found abundantly in the bone marrow [36]. On the other hand, chicken *CATHB1* mRNA shows a more restricted expression pattern, with preferential expression in bursa of Fabricius.

*AvBDs* expression is seen mainly in bone marrow for *AvBD1* through 7, which originate from myeloid cells, and in liver for *AvBD8* through 10 and -13. Other lymphoid tissues did not express *AvBDs* in significant amounts. *AvBD2* and -4 expression is especially very weak and is limited to bone marrow and *AvBD13 is* weakly detected in liver but hardly detected in other tissues even with increased PCR cycles relative to the others. These results are consistent with previous reports [36, 40]. Only *AvBD5* was strongly expressed in thymus. These results show that the tissue specific pattern varies across the *defensin* gene family with some members showing expression in all tested tissues, whereas the majority demonstrate more limited expression patterns which can be divided into three groups. Seven genes (*AvBD1* through *7*) are predominantly expressed in bone marrow, four genes (*AvBD8* through *10* and *13*) are restricted primarily to liver, and three (*AvBD1*, -*12* and -*14*) are expressed in all tested tissues. *NK-lysin* showed strong expression in spleen with intermediate expression in bone marrow, intestine and thymus. Consistent with the role of *cathelicidins*, *defensins* and *NK-lysin* in the first line of host defense, abundant expression of these genes was detected in bursa and bone marrow. The transcriptional regulatory mechanism of these genes during development and under pathogen infection remains to be demonstrated.

The antibacterial efficacy of several *defensins*, *cathelcidins* and *NK-lysin* has been evaluated [2, 9, 23, 36, 40]. Like their mammalian counterparts, most chicken *AvBD*, *CATHs* and *NK-lysin* are capable of killing bacteria. Cuperus et al. reviewed antimicrobial activity of avian HDPs against selected pathogens [41]. Zhang and Sunkara also reviewed expression, antimicrobial and immunomodulatory activities of HDPs, but there are no direct, comprehensive comparisons of major HDPs [30]. Here, we synthesized 19 chicken HDPs and analyzed antibacterial activity against *E. coli* in a comprehensive direct comparison*.* These peptides differ in net charge from 0.1 to 10 and also vary in length from 28 to 44 amino acids. They also vary in expected hydrophobicity from 24 to 61 % (Table 1).

**Table 1:** Sequences and properties of peptides used in this study

Name	Length (aa)	Composition	Mw (g/mol)	Net charge	Hydrophobic ratio
AvBD1	40	GRKSDCFRKSGFCAFLKCPSLTLISGKCSRFYLCCKRIWG	4567.55	7.7	32
AvBD2	39	RDMLFCKGGSCHFGGCPSHLIKVGSCFGFRSCCKWPWNA	4324.14	3.9	36
AvBD3	36	TATQCRIRGGFCRVGSCRFPHIAIGKCATFISCCGR	3877.62	5.8	33
AvBD4	37	RYHMQCGYRGTFCTPGKCPYGNAYLGLCRPKYSCCRW	4341.08	5.8	24
AvBD5	37	GLPQDCERRGGFCSHKSCPPGIGRIGLCSKEDFCCRS	4000.59	1.8	24
AvBD6	38	SPIHACRYQRGVCIPGPCRWPYYRVGSCGSGLKSCCVR	4216.96	5.8	32
AvBD7	37	RPIDTCRLRNGLCFPGICRRPYYWIGTCNNGIGSCCAR	4307.05	4.7	32
AvBD8	41	NNEAQCEQAGGICSKDHCFHLHTRAFGHCQRGVPCCRTVYD	4593.16	0.1	24
AvBD9	42	ADTLACRQSMGSCSFVACRAPSVDIGTCRGGKLKCCKWAPSS	4353.13	3.7	36
AvBD10	42	PDTVACRTQGNFCRAGACPPTFTISGQCHGGLLNCCAKIPAQ	4309.01	1.8	38
AvBD11	39	RDTSRCVGYHGYCIRSKVCPKPFAAFGTCSWRQKTCCVD	4431.14	4.8	28
AvBD12	44	PDSCNHDRGLCRVGNCNPGEYLAKYCFEPVILLCCKPLSPTPTKT	4841.64	0.8	36
AvBD13	37	SDSQLCRNNHGHCRRLCFHMESWAGSCMNGRLRCCRF	4373.03	4	24
AvBD14	37	DTVTCRKMKGKCSFLLCPFFKRSSGTCYNGLAKCCRP	4151	6.7	30
CATH1	28	PVRVKRVWPLVIRTVIAGYNLYRAIKKK	3338.14	8	53
CATB1	40	PIRNWWIRIWEWLNGIRKRLRQRSPFYVRGHLNVTSTPQP	5028.86	7.1	45
CATH2	34	PVLVQRGRFGRFLRKIRRFRPKVTITIQGSARFG	4014.84	10	44
CATH3	31	PVRVKRFWPLVPVAINTVAAGINLYKAIRRK	3547.35	7	61
cNK3	30	PDEDAINNALNKVCSTGRRQRSICKQLLKK	3399.95	3.9	30

Molecular weight, net charge at pH7 and hydrophobic ratio were calculated using Peptide property calculator (http://www.innovagen.proteomics-tools)

Even though many tested chicken HDPs show varying efficiencies against pathogens, the majority kill bacteria at low concentration. AvBD5, -8, -10 and -12 show minimal killing activity among tested HDPs at less than 5 μM. This is consistent with the previous report that AvBD8 activity showed 27 μM as lethal dose (LD)_50_ against *E.coli* [42]. These four peptides have very low net charge from 0.1 to 1.8. AvBD4 has 24 % hydrophobicity with 5.8 net charge and can kill bacteria very well compared to AvBD5, -8 and -13 that have the same hydrophobicity but lower cationicity of 0.1 to 4. This suggests that low net charge results in inefficient antibacterial efficacy, even with a suitable hydrophobicity. However, AvBD3, -4 and -6 have the same net charge (5.8) and these peptides kill effectively over all tested concentrations, although BD4 has 33 % hydrophobicity and the strongest activity among the three. Also, there is 6 % gap in hydrophobicity between AvBD2 and cNK3, which share 3.9 net charge but demonstrate different activity. This result suggests that, hydrophobicity is an important factor in antibacterial activity of peptides. A structural effect cannot be ruled out but this result revealed that antimicrobial activity is strongly influenced by cationicity and hydrophobicity. Although, the *CATH* family has more overall cationicity and hydrophobicity than the *AvBD* family, but this does not translate to higher antibacterial activity. The C-terminus of some of our synthesized peptides was abbreviated relative to natural peptides. We recognize that this could impact cationicity and hydrophobicity on antibacterial activity. This potential discrepancy should be clarified in future experiment.

In the present study, all four CATH*s* reduced *E. coli* cell viability and severely damaged *E. coli* cell membranes, with CATH2 and CATH3 showing the highest efficiency. In previous studies, the variable domains of CATH1, CATH2, and CATH3 were clearly discriminated, but the variable domain of CATHB1 was not identified [35, 36]. Here, we predicted the variable domain sequence of CATHB1 and demonstrated weak antibacterial activity compared to others.

Mechanisms for bacteria killing by β*-defensin* are thought to be similar to those of other cationic HDPs where positively charged residues interact with negatively charged membrane components, after which hydrophobic residues insert into the membrane, disrupting it and killing the cells [43, 44]. It is generally accepted that increasing the hydrophobicity of the nonpolar face of the amphipathic α-helical peptides will also increase the antimicrobial activity [45, 46]. Increased cationicity also helps to enhance antibacterial activity [9]. Cationicity is important for killing bacteria [47], but simply increasing the net charge does not result in the improvement of antimicrobial potency [48]. Hydrophobicity also requires an optimal range to enhance antibacterial activity [45]. Disruption of structural integrity is another important factor for high efficiency in bacteria killing [49, 50]. Our results indicate that the mode of antibiotic action of HDPs requires a balance between cationicity and hydrophobicity to optimize bacteria killing activity. But the relationships between two important factors, cationicity and hydrophobicity, and antimicrobial efficacy of HDPs remains to be determined. Antimicrobial efficacy of 19 peptides against *E.coli* in this study are consistent with the previous report [35]. Future study against other bacteria that cause bacterial disease in birds will help to improve our understanding of the role of these genes in immunity to bacteria in chickens.

## Conclusions

Antimicrobial activity and differential tissue expression patterns of 19 chicken HDPs were analyzed. In summary, we confirmed that most of the HDPs showed antibacterial activity against *E.coli* and demonstrate their differential tissue expression pattern. These studies highlight the dose-dependent antimicrobial effects that were mediated by membrane damage and the importance of balance between cationicity and hydrophobicity. Gene expression of chicken HDPs are variable and the AvBD gene family can be divided into two functional expressional groups.

## Additional file

Additional file 1: Table S1. Primers used in this study. (DOCX 14 kb)

## Abbreviations

AMP: Antimicrobial peptide
HDPs: Host defense peptides
