# Supplementary material for: Tissue expression and antibacterial activity of host defense peptides in chicken
Source: BMC Vet Res. 2016 Oct 13;12:231. doi: 10.1186/s12917-016-0866-6 (PMC5064907; doi:10.1186/s12917-016-0866-6)
Supplement: Additional file 1: Table S1. — Primers used in this study. (DOCX 14 kb) [file 12917_2016_866_MOESM1_ESM.docx]

| Name | Forward | Reverse | Reference |  |
| --- | --- | --- | --- | --- |
| AvBD 1 | GAAAACCCGGGACAGACG | AGCGAGAAGCCAGGGTGAT | Hong et al., 2012 | |
| AvBD 2 | TCTGCAGCCATGAGGATTC | TAAAGCACATGCCTGGAAGAAAT | Hong et al., 2012 | |
| AvBD 3 | ACCCAGTGCAGAATAAGAGGAG | AGACTGCAGAGGTGGGAAGG | Hong et al., 2012 | |
| AvBD 4 | AATCCTTTGCTTTTTCATC | TGCAATTATTCACACTACAACCAT | Hong et al., 2012 | |
| AvBD5 | GTGACCCTCCGGGCATCT | ATGAACGTGAAGGGACATCAGAG | Hong et al., 2012 | |
| AvBD 6 | ATCCTTTACCTGCTGCTGTCTGT | GAGGCCATTTGGTAGTTGC | Hong et al., 2012 | |
| AvBD 7 | GCTGTCTGTCCTCTTTGTGGTG | ATTTGGTAGATGCAGGAAGGAT | Hong et al., 2012 | |
| AvBD 8 | TGTGGCTGTTGTGTTTTGT | CTGCTTAGCTGGTCTGAGG | Hong et al., 2012 | |
| AvBD 9 | ACCGTCAGGCATCTTCACAG | CCATTTGCAGCATTTCAGC | Hong et al., 2012 | |
| AvBD 10 | GAATTGGGGCACGCAGTC | CCGGAATCTTGGCACAGC | Hong et al., 2012 | |
| AvBD 11.4 | CACAGAGAGAGGCAGAAGGT | TGAAGTTTGGAGTGGGAGGG | This study | |
| AvBD 12.3 | AGACAGCTGTAACCACGACA | CTGCAGTTCGGACACCTTCA | This study | |
| AvBD 13.4 | TCGTTGTCATTCTCCTCCTCC | CGTTCATGCAGCTCCCAG | This study | |
| AvBD 14 | ATGGGCATATTCCTCCTGT | CACTTTGCCAGTCCATTGT | Hong et al., 2012 | |
| CATH1 | GCTGTGGACTCCTACAACCAAC | GGAGTCCACGCAGGTGACATC | Achanta et al.,2012 | |
| CATH2 | CAAGGAGAATGGGGTCATCAG | CGTGGCCCCATTTATTCATTCA | Achanta et al.,2012 | |
| CATH3 | GCTGTGGACTCCTACAACCAAC | TGGCTTTGTAGAGGTTGATGC | Achanta et al.,2012 | |
| CATB1 | CCGTGTCCATAGAGCAGCAG | AGTGCTGGTGACGTTCAGATG | Achanta et al.,2012 | |
| NK-lysin | GATGCAGATGAAGGGGACGC | CTGCCGGAGCTTCTTCAACA | Lee et al., 2014 | |
| GAPDH | GGTGAAAGTCGGAGTCAACGG | TCGATGAAGGGATCATTGATGGC | Lee et al., 2014 | |
